# Supplementary material for: The interactome of intact mitochondria by cross-linking mass spectrometry provides evidence for coexisting respiratory supercomplexes
Source: Mol Cell Proteomics. 2017 Dec 8;17(2):216–32. doi: 10.1074/mcp.RA117.000470 (PMC5795388; doi:10.1074/mcp.RA117.000470)
Supplement: Supplemental Data [file supp_RA117.000470_133922_0_supp_23978_fzffwf.zip › spectra_annotation/mito_spectra_annotation/372-1-5-1-1-1.pdf]

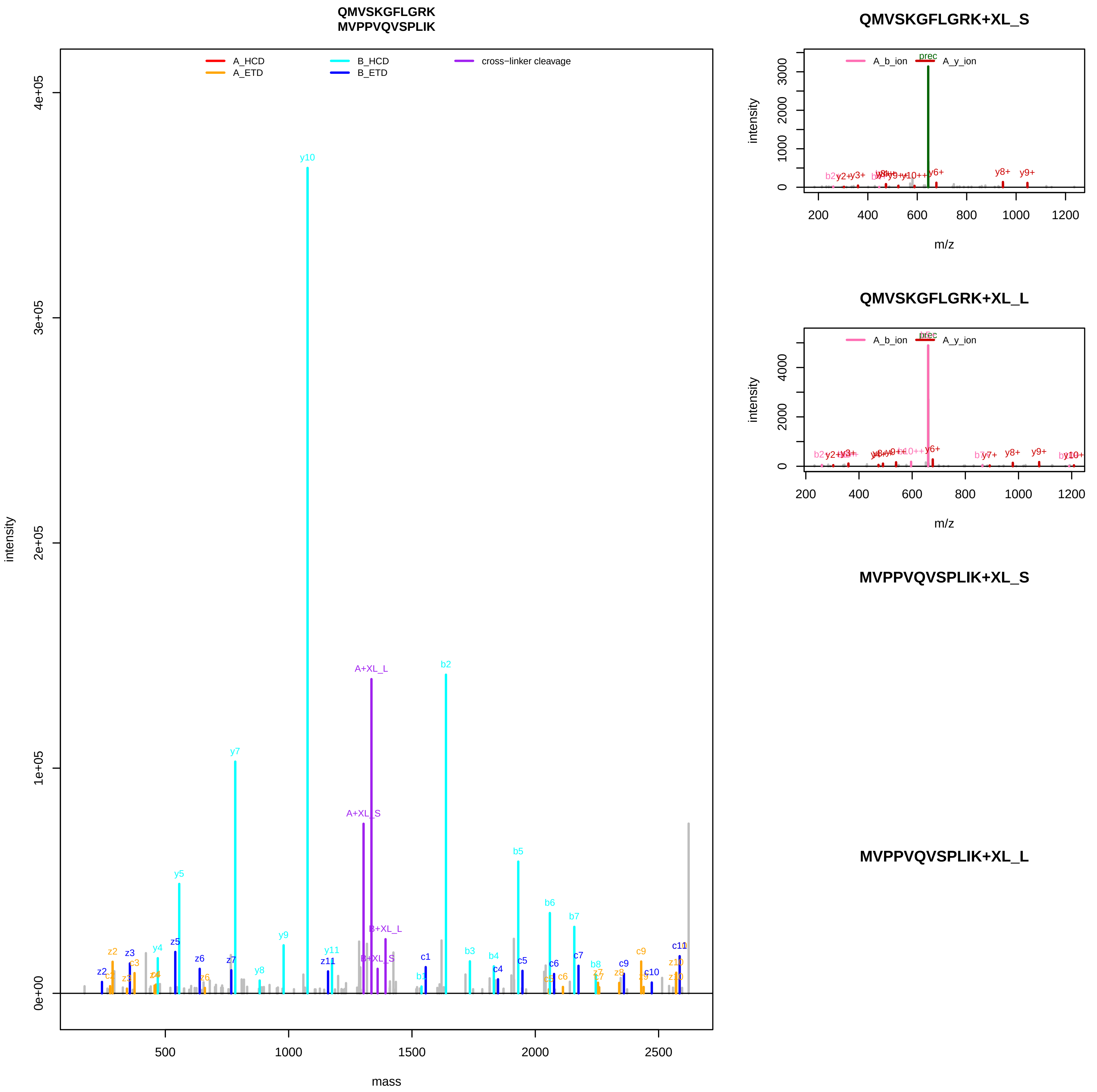
QMVSKGFLGRK
MVPPVQVSPLIK
A_HCD
A_ETD
B_HCD
B_ETD
cross−linker cleavage
intensity
mass
0e+00
1e+05
2e+05
3e+05
4e+05
500
1000
1500
2000
2500
y10
A+XL_L
b2
y7
A+XL_S
b5
y5
B+XL_L
b6
b7
y9
y11
B+XL_S
c11
QMVSKGFLGRK+XL_S
QMVSKGFLGRK+XL_L
MVPPVQVSPLIK+XL_S
MVPPVQVSPLIK+XL_L
A_b_ion
A_y_ion
prec
m/z
y6+
y8+
y9+
